# Real-time tracking of lung tumors using a 1.5T Elekta Unity MR-Linac: first clinical experiences

**DOI:** 10.3389/fonc.2025.1569428

**Published:** 2025-04-08

**Authors:** Blake Smith, Bryan Allen, Samuel Rusu, Joel St-Aubin, Daniel Hyer

**Affiliations:** Department of Radiation Oncology, University of Iowa Health Care, Iowa City, Iowa, United States

**Keywords:** adaptive, MR-Linac, gating, lung, motion management

## Abstract

There is a growing interest in the application of MRI-guided adaptive radiotherapy (MRIgART) to improve the treatment of lung tumors. Motion management plays a central role to better localize these tumors and minimize toxicities to surrounding healthy tissue. Elekta recently released their comprehensive motion management system for the Unity 1.5T MR-Linac, enabling real-time tracking and predictive gating using MR cine imaging during treatment. We report our first clinical experiences using this gating technology to treat patients with central lung tumors adjacent to critical structures on the Unity MR-Linac using both conventional and SBRT fractionations. A surrogate structure was tracked if the primary target could not be visualized. Beam gating was automatically performed if the system detected the target moving outside of the defined gating envelope, which was set as the planning target volume. Integrating this technology has profoundly impacted our MRIgART program; we have been able to treat challenging lung tumor patients with greater precision and have begun to reduce our PTV margins, leading to improved dose sparing of nearby critical structures.

## 1 Introduction

The role of MRI-guided adaptive radiotherapy (MRIgART) has grown throughout the radiotherapy community. During MRIgART, a daily MR image is used to tailor the treatment to the daily anatomy by delineating organs at risk (OAR) as well as the target and re-optimizing the treatment to preserve the nominal planning objectives among each fraction (1). These advantages have proven useful for a multitude of clinical sites including prostate (2), heart (3), spine (4), lung (5, 6), liver and pancreas (7). Recently, the role of MRIgART has been increasing for lung treatments, particularly for SBRT, utilizing fractional adaptation to enable more central SBRT treatments in close proximity to the bronchial tree and other critical structures (8).

The MR-Linac is an ideal technology for the treatment of lung tumors. A recent study from Merckel et al. describes their institution’s initial experiences using the Elekta Unity (Elekta AB, Stockholm, Sweden) to perform non-gated SBRT treatments for ultra-central lung tumors, which demonstrated improved target coverage by adapting the treatment for each fraction (6). MR-Linacs also employ live 2D MR Cine imaging for real-time anatomical monitoring where the target position can be directly visualized during treatment, potentially reducing intrafraction uncertainties. Elekta recently released their comprehensive motion management (CMM) system that enables real-time tracking and gating based on these cine MR images and a predictive algorithm to minimize latency, which has shown promising experimental results (9, 10). Therefore, this work focused on presenting our institution’s first clinical experiences using these new technologies for lung tumors treated on a 1.5T Unity MR-Linac.

## 2 Methods and materials

### 2.1 Patients

Eight lung patients with central lung tumors were treated using the CMM system on the Unity MR-Linac. The patient cohort is summarized in **Table 1**. Patients identified for treatment on the MRLinac had central or ultracentral tumors adjacent to a bronchus, esophagus, great vessel, or heart. Patients identified for SBRT had tumors that were typically less than 5 cm in greatest dimension, no identified mediastinal disease, and were not eligible for surgery or elected not to have surgical resection. This quality assurance and improvement project was reviewed by the IRB of Record (UIOWA IRB-01, biomedical) and deemed not to meet criteria for human subjects research.

**Table 1:** Patient (Pat) selection, disease, and prescriptions treated for gated lung treatments on the Unity MR-Linac.

Pat #	Disease	Location	Rx Dose (cGy)	# Fractions
1	Small Cell Lung Cancer	Posterior to heart	3000	10
2	Renal Cell Carcinoma	Left Hilum	2000	5
3	Squamous Cell Lung Cancer	Right Bronchus	6000	8
4	Adenocarcinoma Lung Cancer	Right Bronchus	6000	8
5	Metastatic Leiomyosarcoma	Lower Left Bronchus	2400	3
6	Adenocarcinoma Lung Cancer	Thoracic Aorta	6000	8
7	Squamous Cell Lung Cancer	Right Hilum	6000	30
8	Adenocarcinoma Lung Cancer	Heart and Liver	6000	8

### 2.2 Pre-treatment planning

Reference plans were created in Monaco version 6.2.1.0 (Elekta AB, Stockholm, Sweden) (11). An exhale breath-hold CT with abdominal compression acquired on a Biograph Vision PET/CT scanner (Siemens, Erlangen, Germany) was used as the primary dataset. Additional datasets from simulation included pre- and post-gadolinium T1-weighted images along with T2-weighted images from a 3T Magnetom Vida MRI (Siemens Healthineers, Erlangen, Germany). Respiratory motion was accounted for by creating an ITV from individual 4DCT phases. Depending on the specific treatment case, PTV margins between 2 and 5 mm were used. Treatment planning objectives were optimized for each plan to maximize the adaptive plan quality and reproducibility based on the specific fractionation and positioning of regional anatomy surrounding the treatment region. In particular, dosimetric planning goals for the SBRT 60 Gy in 8 fraction regiment were referenced from the SUNSET trial (12). All treatments were step-and-shoot IMRT with 9-11 ipsilateral beams. Pre-treatment quality assurance (QA) was performed following the guidelines from AAPM TG 218 (13).

### 2.3 Online workflow and planning

Treatments were adapted from a T1-weighted 3DVaneXD MRI for each fraction. Rigid registration was performed between the reference CT and the daily MRI to evaluate the need for an adaptation. An adapt-to-position (ATP) workflow, which alters the MLC positions and re-weights the segments to reproduce the nominal target coverage to compensate for small deviations in the patient setup, was used when the anatomy matched well with the simulation or previous fraction contours. Due to the limited modifications made to the original treatment plan, this technique is often regarded as the analog of applying couch shifts to correct for setup errors with traditional, non-adaptive external beam therapy. Large anatomical deviations were addressed using an adapt-to-shape (ATS) workflow that consists of generating a new plan tailored to the unique anatomy from the acquired pre-treatment MRI. ATS treatments were re-optimized from fluence to achieve the DVH goals of the reference plan. Adapted treatment plans were not subject to additional measurement-based QA, but rather relied on calculation-based QA (14).

### 2.4 Assessment of online adaptive planning

In order to assess the impact of adaptive radiotherapy for central lung tumors, all patients that received 60 Gy in 8 fractions were retrospectively evaluated with both ATP and ATS treatment plans. This was performed by creating ATP-adapted treatment plans for each fraction of the treatment course in which an ATS was performed clinically. Due to the offline nature of this retrospective evaluation and to provide a direct comparison to the clinical ATS plans, dose calculations were performed on the adapted anatomy with the corresponding bulk density assignments. All plans were normalized to the prescription coverage specified by the ATS-adapted clinical treatment plan and DVH deviations from the reference plan were recorded for the conformity index (CI) of the target and maximum dose for the spinal cord, heart, esophagus, bronchus, and aorta.

### 2.5 Impact of bulk density assignment

Bulk electron densities are used for ATS online dose calculations. Due to the heterogeneity of lung tissue, the accuracy of using bulk densities for lung treatments was evaluated. Following the initial reference plan generation, the plans were recalculated using the bulk density assignments and the target coverage, homogeneity index, max dose and Paddock conformity were evaluated.

### 2.6 Motioning monitoring and beam gating

Gating on the Elekta Unity is enabled through the CMM system that integrates live 2D imaging to automate beam gating decisions from a balanced turbo field echo (bTFE) MR cine (5 Hz) captured in alternating coronal and sagittal planes. Gating decisions were determined in the anatomical position monitoring (APM) system based on the overlap of the target structure (often the GTV) relative to the gating envelope (set as the PTV for this study). The CMM respiratory gating techniques utilizes an algorithm that predicts the position of a target to compensate for the system latency. The specific technique used in this work was *VOICE (Volume Overlapping Criterion)*, where the beam gates off if the overlap of the target and gating envelope is expected drop below a user-defined criteria (typically 95% at our institution). A surrogate structure tracking technique that we have developed for the Elekta Unity was utilized for some cases to improve tracking accuracy or if multiple targets were treated simultaneously (15).

## 3 Results

**Figure 1:**
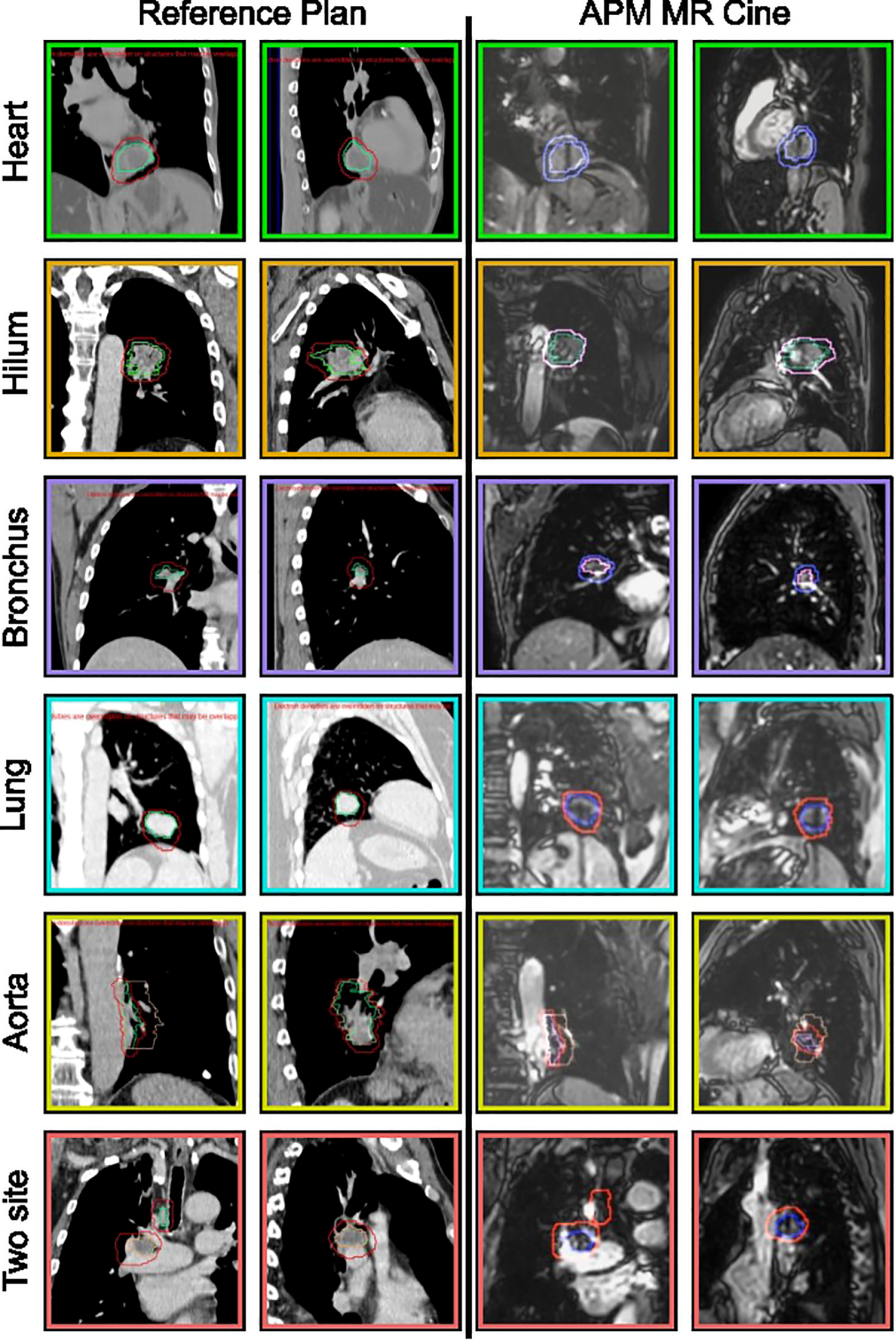
Subset of lung sites treated using MR cine gating on the Elekta Unity MRLinac. The reference CT is shown in columns 1 and 2 with the coronal and sagittal views of the target, green, gating envelope, red, and surrogate (peach, if applicable). Columns 3 and 4 are the MR cine analogs of the reference plan showing the tracking structure, surrogate (if used), and gating envelope.

**Figure 2:**
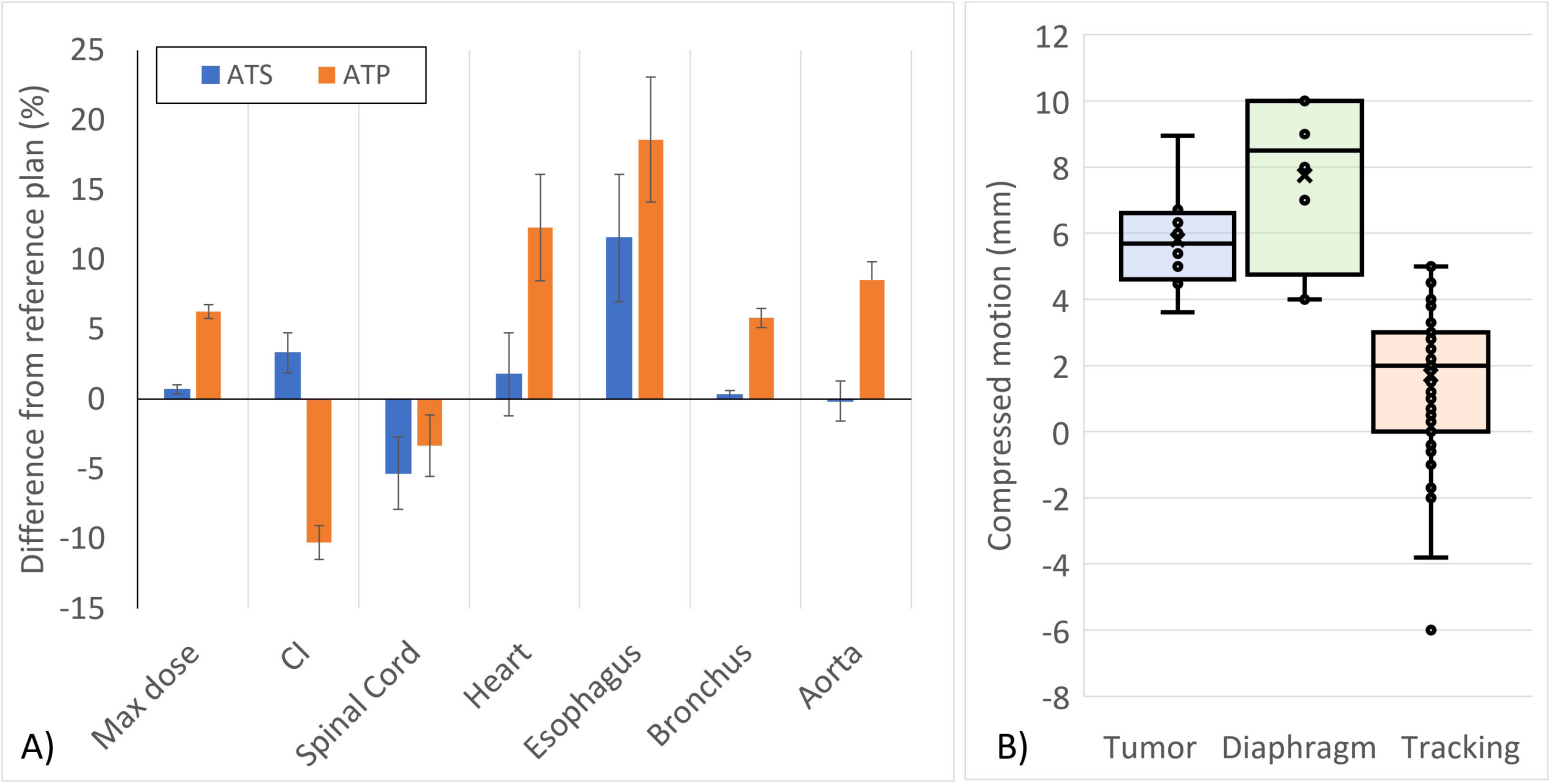
**(A)** Deviations from the reference planning DVH objective constraints for online ATS-adapted and retrospective ATP-planned SBRT treatments for lung tumors. Error bars represent the k = 1 standard error of the mean. **(B)** Distribution of tumor motion with abdominal compression. Compression constancy was tracked for each fraction by comparing the diaphragm motion at the time of simulation against the diaphragm motion measured using a 2D cine prior to treatment.

The treatment DVH criteria are summarized in **Table 2** and the tracking strategies are illustrated in **Figure 1**. The average compressed tumor motion for the subset of patients was 5.8 mm with the distribution shown in **Figure 2**. Constancy of diaphragm compression, based on the monitored motion of the diaphragm, was on average within 1.7 mm of baseline established at the time of simulation. Direct tracking of the intended target was performed for patients 1-5, whereas a surrogate structure was used for patients 6 - 8. The surrogate structure for patient 6 was defined as a 1.5 cm lateral expansion of the GTV into the ipsilateral lung from the aorta. Treatments planned for patients 7 and 8 consisted of two targets with a single isocenter where one of the targets was selected to be tracked while a slightly larger PTV margin was added to the non-tracked target. The targets for patient 7 were grouped as a single target for dose reporting.

**Table 2:** Treatment metrics between plans calculated on the reference CT and bulk relative electron density (E.D.) assignment dataset, which includes the target coverage (cov), plan maximum (Max) dose, conformity index (CI), and homogeneity index (H.I.).

Pat #	Target		E.D. Overrides		Reference (CT) plan dosimetry				Bulk Density dosimetry				Scale
	#	Vol (cc)	Target	Lung	Cov	Max (cGy)	CI	HI	Cov	Max (cGy)	CI	HI	Factor
1	1	57.9	1.04	0.19	0.99	2171	0.83	1.04	1.00	2237	0.83	1.04	0.991
2	1	75.8	1	0.29	0.97	2197	0.89	1.05	0.95	2236	0.87	1.07	1.010
3	1	12.2	0.98	0.25	0.95	6638	0.83	1.07	0.86	6618	0.83	1.10	1.027
4	1	7.4	0.98	0.16	0.95	6619	0.80	1.08	0.96	6954	0.80	1.10	0.991
5	1	22.1	1.05	0.41	0.98	3032	0.85	1.18	0.97	3071	0.83	1.19	1.007
6	1	24.5	1.07	0.23	0.95	6582	0.69	1.06	0.92	6677	0.69	1.08	0.998
7	2	49.7	1.06	0.24	0.95	6690	0.77	1.08	0.91	6714	0.77	1.08	1.011
8	2	28.1	1.05	0.28	0.96	7359	N/A	1.18	0.95	7431	N/A	1.20	1.000
8		10.6	1.03		0.66			1.54	0.62			1.53	1.011

NA, Not Applicable.

The impact of bulk density assignment for the lung is summarized in **Table 2**. While the lung is heterogeneous, only small differences were observed between the nominal plan calculated on the CT dataset and the bulk density dataset.

When evaluating the impact of adapting treatments retrospectively, notable deviations from the reference plan were observed without daily adaptation. The plot in **Figure 2** shows the average DVH criterion differences among the ATS-adapted and retrospectively planned ATP treatments relative to the baseline DVH criterion values from their respective reference treatment plans. With the exception of one instance, where the deviation from the DVH goal was within the uncertainty of the Monte Carlo calculation, all clinically delivered ATS fractions met the DVH goals from the reference plan. In some instances, the ATS plan improved upon the reference plan as shown in the general trend observed for the conformity index and spinal cord doses. In nearly all ATP cases, the CI was degraded relative to the reference plan.

## 4 Discussion

Comprehensive Motion Management coupled with MRIgART ensures that the intended target is correctly treated throughout treatment. The direct tracking of these targets, rather than utilizing external surrogates, enable confidence for clinicians to treat central and ultracentral lung tumors safely. In many cases, direct tracking of the tumor was utilized for the patients presented in this study. However, target locations near the great vessels were observed to have issues with tracking due to the very high contrast of blood flow on the bTFE MR Cine sequence (e.g., tumors directly adjacent to the heart or thoracic aorta). Expanding the GTV 1-2 cm into the adjacent lung tissue and away from these hyperintense objects created successful tracking surrogates for these cases. For all patients, the total motion of the targets was reduced through the use of abdominal compression.

Multiple motion management strategies are utilized to reduce treatment volumes in the cohort of presented patient treatments. Abdominal compression was successfully used to minimize the total respiratory motion of the target to within an average displacement of 5.7 mm whereas the active monitoring and respiratory gating capabilities with CMM enable greater localization of the treatment by directly tracking the GTV or an internal surrogate and monitoring its spatial position relative to the intended treatment volume. As such, ITV to PTV margins of as little as 2 mm were successfully used with high treatment efficiency. Previous work at our institution has studied and developed multiple tracking techniques for both direct and surrogate tracking, resulting in tracking accuracy rates of 98.9% throughout the entire course of treatment (15). While CMM offers active gating strategies that can solely achieve similar treatment volumes, abdominal compression provides an additional benefit of an improved treatment efficiency as the total motion is minimized, which improves duty cycle. Prior gating studies performed during the commissioning of CMM at our institution have shown that the gating using predictive respiratory modeling adequately mitigates system latency to provide nearly the same dose distribution achievable from a system without latency (10).

The integrated use of MR imaging for adaptive planning and cine motion monitoring for the Elekta Unity demonstrates high geometric fidelity, which is a critical feature for MRIgART systems to deliver SBRT treatments that rely on precise localization and small PTV margins. Specifically, the magnitude of magnetic field inhomogeneity and RF interference has been shown to be minimal for the Elekta Unity (11, 16). The initial commissioning of the Elekta Unity at our clinic quantified the magnitude of image distortion to be under 1 mm in any direction over the range of diameter spherical volumes up to 400 mm and within 0.4 mm within a 200 mm diameter spherical volume (11), the latter of which corresponds to the region where most treatment volumes are displaced from the imaging isocenter on the Unity MRLinac. As such, MR distortion is expected to have a negligible impact on treatment accuracy given that nearly all adaptive treatments on the Unity MRLinac are registered and anatomically aligned within a 200 mm diameter spherical volume to isocenter. Within this region, geometric distortions that are less than the size of a voxel will have a negligible dosimetric impact. However, in the effort to further reduce treatment margins using MRIgART, it is important to note that geometric distortion, in addition to the error in the MR-to-MV isocenter coincidence, will impact the minimum theoretical PTV margin to account for the uncertainty in the MR image fidelity in the absence of any other geometric uncertainty.

In the cases presented, online ATS planning improved the conformity index and the maximum spinal cord dose relative to the reference plan with some minor variations in the maximum dose to the PTV, heart, bronchus, and aorta. On average, non-adaptive treatments resulted in higher doses to these structures due to their close proximity to the treatment region. However, both the clinically delivered ATS and retrospectively analyzed ATP treatments resulted in higher esophagus doses. This was due to the fact that the maximum doses to other critical structures in closer proximity to the PTV were prioritized over the esophagus. While the dose to the esophagus was elevated, there were no instances where the maximum dose exceeded its corresponding DVH goal.

Subtle changes occurred to the target coverage, conformity, and heterogeneity due to bulk density assignments. Upon initial inspection, a notable change occurred to the target coverage between datasets for patient 3 in **Table 2** (a coverage reduction from 95% to 86%). A factor of 1.027 was necessary to rescale this bulk-density calculated plan back to the original coverage, indicating that the coverage difference is more driven by changes in the resulting target dose than changes to the targeted dose distribution. It should also be noted that a 2.7% dose is within the standard daily operational tolerances for therapeutic linacs (17).

## 5 Conclusions

MR cine guided gating treatments for lung tumors are demonstrated using the CMM on the Elekta Unity 1.5T MR-Linac. Since its incorporation, our MRIgART service has played a complementary role within our department to treat challenging lung tumors near critical structures. Future work and development will further reduce margins and improve our gating strategies to enhance healthy tissue sparing.

## Data Availability

Data sharing is not available. Requests to access the datasets should be directed to blake-smith@uiowa.edu.
